# Acute Myeloid Leukemia (AML) with Erythroid Predominance Exhibits Clinical and Molecular Characteristics that Differ from Other Types of AML

**DOI:** 10.1371/journal.pone.0041485

**Published:** 2012-07-23

**Authors:** Zhuang Zuo, L. Jeffrey Medeiros, Zhao Chen, Dingsheng Liu, Carlos E. Bueso-Ramos, Rajyalakshmi Luthra, Sa A.Wang

**Affiliations:** Department of Hematopathology, The University of Texas M. D. Anderson Cancer Center, Houston, Texas, United States of America; Emory University, United States of America

## Abstract

The clinical importance of erythroid predominance in bone marrow of patients with acute myeloid leukemia (AML) is controversial. These cases represent a heterogeneous group of diseases that historically have been classified into different categories. We studied 313 AML patients and specifically compared the clinical, cytogenetic, and molecular features of cases of AML with erythroid predominance, arbitrarily defined as ≥50% erythroid precursors, to AML cases without erythroid predominance. We also assessed 51 patients with a high-grade myelodysplastic syndrome (MDS), refractory anemia with excess blasts (RAEB). All neoplasms were classified according to the World Health Organization classification. With the exception of therapy-related AML/MDS, the presence of erythroid predominance in variously classified categories of AML was associated with a survival advantage. In addition, AML with erythroid predominance had a lower frequency of cytogenetic abnormalities as well as a lower frequency of mutations involving *NPM1*, *NRAS* and *FLT3* as compared with AML without erythroid predominance. We conclude that the clinical, cytogenetic, and molecular features of AML with erythroid predominance in the non-therapy-related setting are much closer to those of a high-grade myelodysplastic syndrome than they are to other types of AML.

## Introduction

The definition of myeloid neoplasms with erythroid predominance has been controversial since the inception of this concept about a century ago [1], [2], [3]. Due to a lower number of myeloblasts as a percentage of total nucleated cells, these cases were usually classified as a myelodysplastic syndrome (MDS) in the first edition of French-American-British (FAB) classification system. In 1985, the FAB readdressed the issue by calculating myeloblasts as a percentage of the non-erythroid elements in neoplasms with ≥50% erythroid precursors [1]. Using this approach, myeloid neoplasms with ≥30% blasts were classified as AML of M6 type, whereas myeloid neoplasms with fewer myeloblasts were classified as myelodysplastic syndrome (MDS) with “a major erythroid component”. The current 2008 World Health Organization classification relies on a combination of clinical, morphologic, immunophenotypic, genetic and other biologic features to define specific disease entities [4]. Patients with a history of MDS, marked morphologic evidence of dysplasia, or MDS-related cytogenetic changes are considered to have AML with myelodysplasia-related changes (AML-MRC); and patients with AML and prior cytotoxic exposure are classified as therapy-related myeloid neoplasms (t-AML/MDS) [4]. Using this new classification paradigm, many but not all myeloid neoplasms with erythroid predominance are no longer classified as AEL, and instead are reclassified as MDS, AML-MRC, or t-AML/MDS. However, except for t-AML/MDS, the basis for classifying these cases largely relies on counting blasts and erythroid precursors, and calculating the blast percentage, a somewhat arbitrary approach. As a result of the overwhelming erythroid component, a slight difference in blast counts can lead to a neoplasm being assigned to a different category. In order to better define myeloid neoplasms with erythroid predominance, several groups have studied the clinicopathologic characteristics of this group of diseases and found that blast counts and their specific classifications were irrelevant to patient survival [5], [6], [7], [8]. Instead, cytogenetic data appear to be the best means of risk stratification. Based on these findings, some authors have proposed combining AEL and AML-MRC cases with erythroid predominance into a separate category designated as “acute myeloid leukemia with increased erythropoiesis” [5], [7]. These neoplasms as a group, however, have not been compared with their more common AML counterparts in which erythroid precursors are not predominant.

Previous studies of myeloid neoplasms with erythroid predominance have shown that there are no specific cytogenetic abnormalities that are unique to these diseases [5], [7], [9]. It has been hoped that advances in molecular and genetic research would unveil unique features of AML associated with erythroid predominance. As these cases are rare, however, only a few studies have examined the molecular features of AML or MDS with erythroid predominance, and only in part [5], [7], [10].

In this study we have focused on patients with AML associated with erythroid predominance, to address the clinical characteristics and molecular genetic features that distinguish this group from patients with AML without erythroid predominance. We studied 313 AML patients and compared cases with erythroid predominance to cases in which erythroid precursors were not predominant.

## Methods

### Patients

We searched the archives of The University of Texas MD Anderson Cancer Center (MDACC) between 2000 and 2010 for cases of AML or MDS with erythroid predominance, defined as erythroid precursors ≥50% of total nucleated bone marrow cells, as determined by counting cells on bone marrow aspirate smears. Available bone marrow slides of cases with erythroid predominance were retrieved and reviewed and these cases were classified using the 2008 WHO system into the following categories: AEL, AML-MRC, t-AML/MDS, or refractory anemia with excess blasts (RAEB). Specifically, AEL cases had both erythroid precursors ≥50% of total nucleated bone marrow cells and myeloblasts ≥20% of non-erythroid cells. Cases were classified as AML-MRC if the bone marrow had myeloblasts ≥20% of total nucleated cells and there was a prior diagnosis of MDS, or MDS-related cytogenetic changes, or substantial dysplasia (≥50% cells in at least two lineages). Cases were classified as t-AML/MDS if the patient had a history of malignant neoplasm that had been treated. In cases of RAEB, the myeloblasts were enumerated as a portion of total nucleated cells. Cases diagnosed as myelodysplastic/myeloproliferative neoplasm (MDS/MPN) were not included in this study. Patients who had received stem cell transplantation were also excluded as the therapy is a confounding factor that affects survival.

A major focus of this study was the molecular characteristics of these neoplasms. In recent years (2008 until the present), virtually all new AML cases at MDACC have been tested for *NPM1*, *FLT3*, *RAS*, and *KIT* gene mutations as part of the routine work-up. Some specimens with erythroid predominance were tested specifically for this study if DNA samples were available. For comparison, we also retrieved cases of RAEB and AML without erythroid predominance with mutation data available from 2008 to 2010. As the definition of cases of AML with recurrent cytogenetic abnormalities depends on the presence of the cytogenetic abnormality, and does not discriminate cases with or without erythroid predominance; these cases were not included in this study.

The results of conventional cytogenetics and molecular testing as well as clinical follow-up information were retrieved from the medical records. This retrospective analysis was approved by the Institutional Review Board of MDACC and conducted in accordance with the Declaration of Helsinki. Written consent was given by the patients for their information to be stored in the hospital database and used for research.

### NPM1, FLT3, RAS, and KIT Mutation Analysis

*NPM1* mutations were detected using primers designed to amplify mutational hotspots spanning codons 956–971 of exon 12, followed by capillary electrophoresis as described previously [11]. 200 ng of genomic DNA were amplified with the following PCR conditions: initial denaturation at 95°C for 10 minutes followed by 40 cycles of 30 seconds at 95°C, 30 seconds at 55°C, and 30 seconds at 72°C. A final extension step at 72°C for 7 minutes was then performed. Unpurified fluorescently-labeled PCR products were then loaded onto an ABI Prism 3100 Genetic Analyzer (Applied Biosystems) for electrophoresis.

The *FLT3* internal tandem duplication (*FLT3*-ITD) and tyrosine kinase domain codon 835/836 point mutations (*FLT3*-D835) were detected by a fluorescent-based multiplex PCR assay followed by capillary electrophoresis.[12] For *FLT3*-D835 point mutation analysis, PCR products were digested with *Eco*RV before capillary electrophoresis.

*K-RAS* and *N-RAS* mutations at codons 12,13 and 61 were tested using PCR followed by pyrosequencing as described previously [13]. Mutations in exons 8 and 17 of the *KIT* gene were detected by using Sanger sequencing [11].

### Statistical Methods

Fisher’s exact test or the Chi-square test was applied to categorical variables. The Mann-Whitney test was used for numerical comparisons between groups. Differences among groups were considered significant if *P*-values were <0.05 in a two-tailed test. The probability of overall survival (OS) was estimated with the Kaplan-Meier method. The log-rank test was used to compare risk factor categories in survival analysis. Multivariate analysis was performed by Cox proportional regression model to examine the relationship between OS and patient characteristics. The significant factors were identified by Wald backward stepwise elimination.

## Results

### Study Group

The study group included 313 cases of AML and 51 cases of RAEB that met the inclusion criteria for this study. A total of 106 cases were associated with ≥50% erythroid precursors. Each case was reviewed and reclassified according to the 2008 WHO classification scheme. The cases with erythroid predominance included 59 cases of AEL, 24 cases of AML-MRC, 23 cases of t-AML/MDS, and 23 cases of RAEB. In the 59 cases of AEL, 43 (73%) cases had myeloblasts ≤20% of total nucleated cells but ≥20% non-erythroid cells. The remaining 16 (27%) cases of AEL had myeloblasts ≥20% of total nucleated cells, but were not associated with other features necessary to be classified as AML-MRC (e.g. history of MDS, ≥50% of cells with dysplasia in ≥2 lineages, or MDS-associated cytogenetics changes). All other cases in this study were not associated with erythroid predominance and included 57 cases of AML-MRC, 150 cases of t-AML/MDS, and 28 cases of RAEB.

Table 1 summarizes the characteristics of the patients in each diagnostic group. No significant difference in age and gender was found among the diagnostic groups or between cases with or without erythroid predominance. Cases in the group with erythroid predominance had a lower number of myeloblasts (*P* = 0.042) in the bone marrow.

**Table 1:** Characteristics of Acute Myeloid Leukemia Patients at Time of Initial Diagnosis.

Characteristic	AEL		AML–		MRC					t-AML		/t-MDS				
	(N = 59)	with EP (N = 24)	w/o EP (N = 57)		*P* *			Total (N = 81)	with EP (N = 23)	w/o EP (N = 150)		*P* *			Total (N = 173)	*P* †
Age, year (range)	64 (18–89)	67 (24–86)		66 (20–96)		0.487	66 (20–96)		68 (21–84)		65 (22–84)		0.392	67 (21–84)		0.219
Male/female (ratio)	44/15 (2.9)	14/10 (1.4)		36/21 (1.7)		0.186	50/31 (1.6)		17/6 (2.8)		76/74 (1.0)		0.078	93/80 (1.2)		0.056
Bone marrow aspirate cell count, median % (range)																
myeloblast	14 (5–35)	20 (20–35)		32 (20–90)		0.269	27 (20–90)		12 (4–25)		33 (17–98)		0.010	31 (4–98)		0.042
erythroid precursor	58 (50–85)	53 (50–75)		15 (0–35)		<0.001	22 (0–75)		63 (50–81)		12 (0–43)		<0.001	16 (0–81)		<0.001
myeloblast ofnon-erythroid cells	36 (20–81)	50 (40–80)		N.A.		N.A.	N.A.		31 (20–53)		N.A.		N.A.	N.A.		N.A.
Cytogenetic findings, number of patients (%)																
normal karyotype	19 (32.2)	11 (45.8)		20 (35.1)		0.177	31 (38.3)		9 (39.1)		23 (15.3)		0.223	32 (18.5)		0.012
complex karyotype#	33 (55.9)	9 (37.5)		27 (47.4)		0.432	36 (44.4)		10 (43.5)		73 (48.7)		0.802	83 (48.0)		0.242
Cytogenetic risk by revised UKMRC criteria, number of patients (%)																
Intermediate	24 (40.7)	12 (50.0)		23 (40.4)		0.054	35 (43.2)		11 (47.8)		49 (32.7)		0.011	60 (34.7)		0.068
Unfavorable	35 (59.3)	12 (50.0)		34 (59.6)		0.468	46 (56.8)		12 (52.2)		101 (67.3)		0.165	113 (65.3)		0.212
Common mutations, number of patients (%)																
* NPM1* mutated	1 (1.6)	0 (0)		12 (21)		0.005	12 (14)		0 (0)		7 (4.7)		0.363	7 (4.0)		0.019
* FLT3* mutated	3 (4.7)	3 (10)		9 (16)		0.369	12 (14)		0 (0)		18 (12)		0.272	18 (10)		0.118
* NRAS* mutated	1 (1.7)	3 (10)		13 (23)		0.132	16 (19)		0 (0)		13 (8.7)		0.432	13 (7.5)		0.008
* KIT* mutated	0 (0)	0 (0)		0 (0)		1.000	0 (0)		0 (0)		0 (0)		1.000	0 (0)		1.000

* comparing between cases with and without EE;

† comparing between diagnostic groups;

# ≥3 cytogenetic aberrations.

N.A., not applicable; w/o, without; AEL, acute erythroid leukemia; AML-MRC, AML with myelodysplasia-related changes; EP, erythroid predominance; UKMRC, UK Medical Research Council.

### Cytogenetic Comparison

Cytogenetic risk was categorized according to the revised UK Medical Research Council (UKMRC) criteria for AML [14]. When cases with or without erythroid predominance are combined, t-AML/MDS cases were associated with a higher frequency of abnormal karyotypes (*P* = 0.012), as compared with other groups (Table 1), especially AML-MRC cases (*P* = 0.001). A monosomal karyotype was observed in a subset of cases in all AML groups: AEL, 25 (42%), AML-MRC, 27 (33%), and t-AML/MDS, 79 (46%), with no statistically significant difference (*P* = 0.173). When all AML cases were grouped according to the presence or absence of erythroid predominance, patients with erythroid predominance had a lower frequency of cytogenetic abnormalities (*P* = 0.004), and tended to have a lower cytogenetic risk (*P* = 0.061) (Table 2). These findings were also observed within each WHO diagnostic category: cases with erythroid predominance had lower cytogenetic risk (*P* = 0.011) within the t-AML/MDS group and similarly within the AML-MRC group (*P* = 0.054) (Table 1).

**Table 2:** Comparison of Cases of Acute Myeloid Leukemia (AML) and Refractory Anemia with Excess Blasts (RAEB) with or without Erythroid Predominance (EP).

	AML with EP	AML w/o EP	*P*	RAEB with EP	RAEB w/o EP	*P*
	N = 106	N = 207		N = 23	N = 28	
Median age, year (range)	64 (18–89)	65 (20–96)	0.473	64 (41–82)	70 (49–82)	0.145
Normal karyotype, n (%)	39 (37)	43 (21)	0.004	10 (43.5)	11 (40.7)	1.000
Complex karyotype, n (%)	52 (49)	100 (48)	0.995	9 (39.1)	7 (25.9)	0.373
Cytogenetic risk category, n (%) by revised UKMRC criteria for AML; by IPSS for MDS						
Intermediate	47 (44.3)	72 (34.8)	0.061	12 (52.2)	20 (74.1)	0.108
Unfavorable	59 (55.7)	135 (65.2)	0.110	11 (47.8)	7 (25.9)	0.141
*NPM1*,n (% in all cases; % in normal karyotype cases)	1 (0.9; 2.6)	19 (9.2; 44)	0.010	0 (0;0)	0 (0;0)	1.000
*FLT3,* n (%)	6 (5.7)	27 (13.0)	0.069	1 (4.3)	1 (3.6)	1.000
*RAS,* n (%)	4 (3.8)	26 (12.6)	0.022	0 (0)	4 (14.3)	0.117

UKMRC, UK Medical Research Council; IPSS, International Prognostic Scoring System.

Overall, the frequency of a normal karyotype in patients with AML associated with erythroid predominance was comparable to that of RAEB patients (*P* = 0.768, or *P* = 0.587 in non-therapy related setting). This was true even though a complex karyotype was less frequent in the RAEB group (*P* = 0.045) (Table 3). By contrast, abnormal cytogenetics was significantly more common in patients with AML without erythroid predominance (*P* = 0.006). A monosomal karyotype was observed in 14 (27%) RAEB cases, with no statistical significance of erythroid predominance (*P* = 0.353). The cytogenetic risk of patients with AML versus RAEB was difficult to compare as the latter is classified using the International Prognostic Scoring System (IPSS).

**Table 3:** Comparison of Cytogenetic and Molecular Findings in Non-therapy Related Acute Myeloid Leukemia (AML) with Erythroid Predominance (EP) and Refractory Anemia with Excess Blasts (RAEB).

	AML with EP	RAEB	*P*
	N = 83	N = 51	
Median age, year (range)	64 (18–89)	66 (41–82)	0.145
Normal karyotype, n (%)	30 (36.1)	21 (41.2)	0.587
Complex karyotype, n (%)	42(50.6)	16 (31.4)	0.045
*NPM1*,n (% in all cases; % in normal karyotype cases)	1 (1.2; 3.3)	0 (0;0)	0.434
*FLT3,* n (%)	6 (7.2)	2 (3.9)	0.683
*RAS,* n (%)	4 (4.8)	4 (7.8)	0.733

### Molecular Studies

The gene mutation frequencies in patients with myeloid neoplasms with or without erythroid predominance are summarized in Table 2. In AML, cases with erythroid predominance had a significantly lower frequency of *NPM1* mutations (*P* = 0.010). Particularly, in patients with a normal karyotype, the *NPM1* mutation frequency in cases without erythroid predominance was 44%, in keeping with the reported 50–60% reported frequency in literature [15], [16]. In contrast, only 1 (2.6%) patient with AML with erythroid predominance had an *NPM1* mutation. This patient was a 63-year-old Hispanic man. Bone marrow aspirate smears showed 74% erythroid precursors with dysplastic features and 10% myeloblasts, representing 38% of all non-erythroid elements. The patient had a normal karyotype and no other genes tested were mutated.

Similarly, patients with AML with erythroid predominance had a lower frequency of *RAS* mutations than AML cases with <50% erythroid precursors (4% versus 20%; *P* = 0.022). *FLT3* mutations also appear to be less frequent in AML with erythroid predominance, but this did not achieve statistical significance (5.7% versus 13.0%; *P* = 0.069). *KIT* mutations were not identified in any of the patient groups in this study.

In aggregate, the mutation frequencies detected in patients with AML associated with erythroid predominance were comparable to those of patients with RAEB (Table 3), in particular, RAEB with erythroid predominance (Table 2).

### Survival Studies

**Figure 1:**
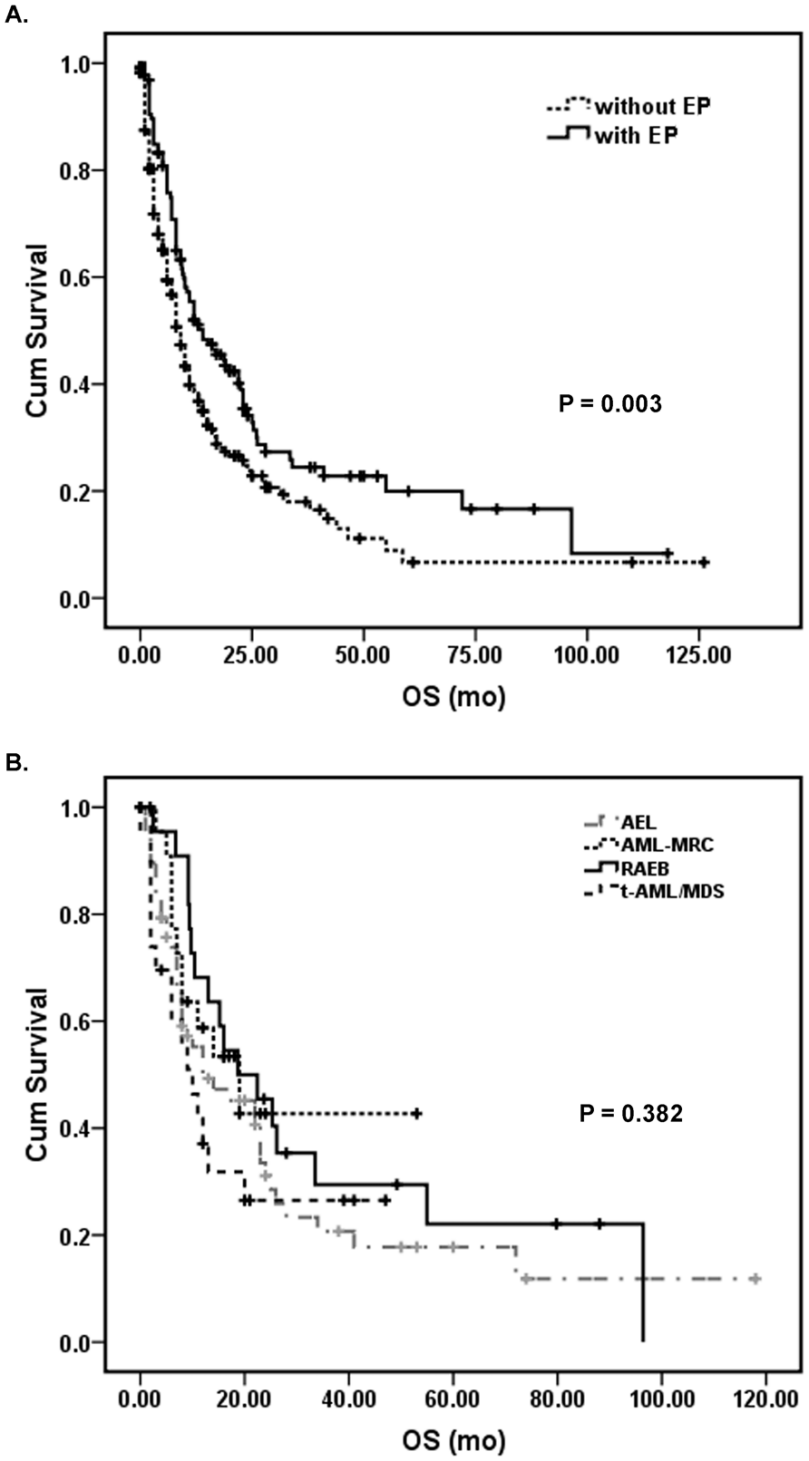
Effects of the presence of erythroid predominance (EP) and diagnostic categories on overall survival. (A) Overall survival of patients in this study cohort stratified by the presence or absence of EP. (B) Overall survival of patients with erythroid predominance stratified by their diagnostic categories.

**Figure 2:**
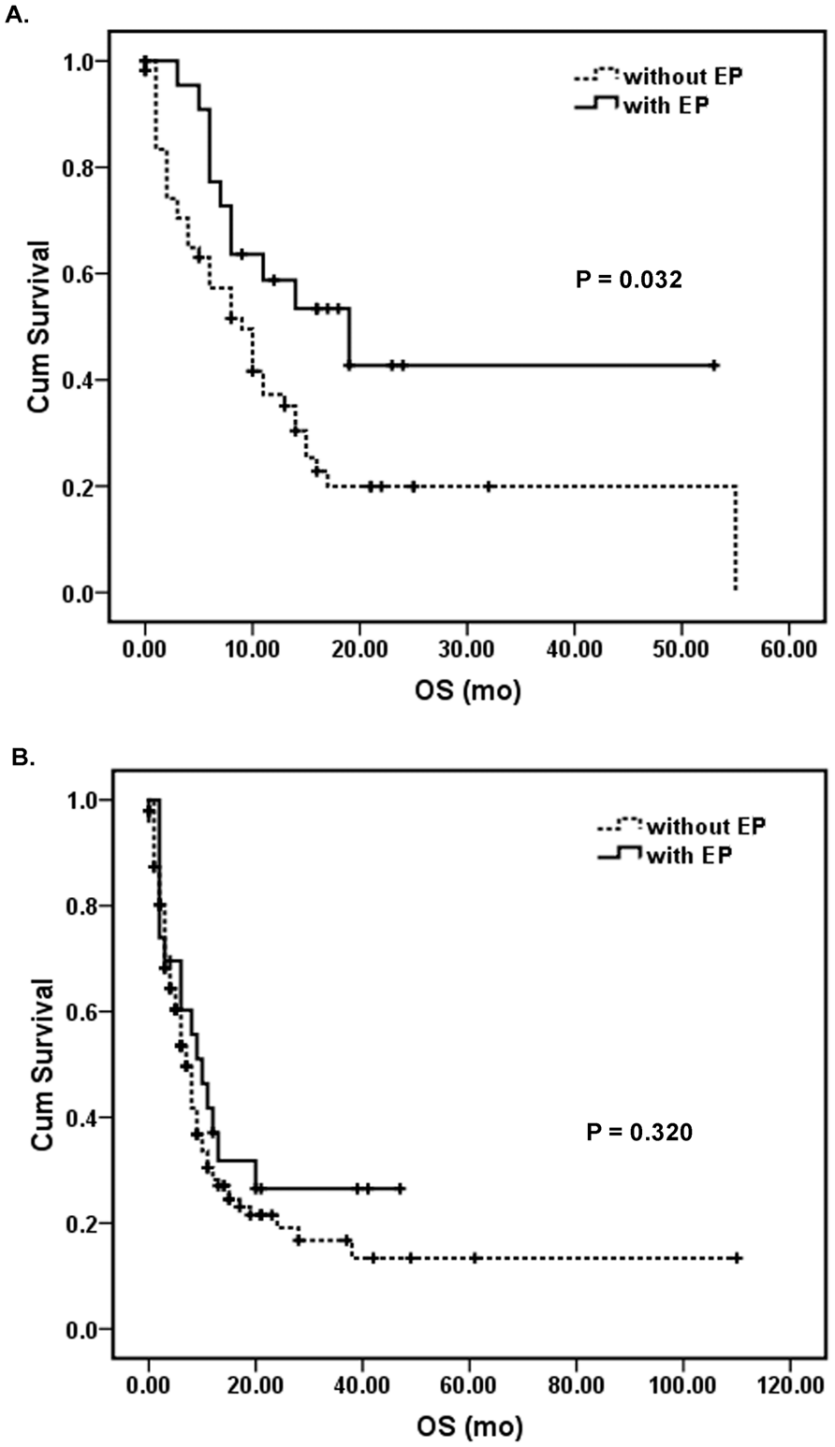
The presence of erythroid predominance (EP) is associated with better overall survival in AML-MRC patients, but not in t-AML/MDS patients. (A) Overall survival of patients with AML-MRC stratified by the presence or absence of EP. (B) Overall survival of patients with t-AML/MDS stratified by the presence or absence of EP.

We stratified the study cohort by the presence or absence of erythroid predominance and compared clinical outcomes. Overall, patients with myeloid neoplasms associated with erythroid predominance had significant better overall survival than patients without erythroid predominance (*P* = 0.003; Figure1A). However, within the group of patients with ≥50% erythroid precursors, the specific diagnostic categories of RAEB, AEL, AML-MRC, or t-AML/MDS had no significant impact on overall survival (*P* = 0.382; Figure 1B). When the presence or absence of erythroid predominance was compared within each diagnostic category, ≥50% erythroid precursors was associated with better overall survival in the AML-MRC group (*P* = 0.032; Figure 2A), but was not significant within the t-AML/MDS (*P* = 0.320; Figure 2B) or RAEB (*P* = 0.909) groups.

To identify independent predictive factors for patient survival, we performed multivariate analysis in AML patients (Table 4). A complex karyotype (*P* = 0.013), unfavorable risk karyotype (*P* = 0.019), age (*P* = 0.002), and the diagnosis of t-AML/MDS (*P* = 0.027) were independent factors for predicting inferior overall survival. Bone marrow myeloblast count as a percentage of total nucleated cells was a borderline hazard (*P* = 0.064) for overall survival. In contrast, the presence of erythroid predominance, and mutation status of genes tested did not significantly predict overall survival.

**Table 4:** Multivariate Analysis for Factors Predictive of Overall Survival.

Variable	Hazard		95% confidence	interval
	ratio	*P*	Lower	Upper
Age at diagnosis	1.025	0.002	1.009	1.042
Gender (male vs female)	0.972	0.636	0.791	1.255
Diagnosis (AML-MRC)	0.729	0.381	0.360	1.478
Diagnosis (t-AML/MDS)	2.109	0.027	1.088	4.086
Bone marrow myeloblasts	1.014	0.064	0.999	1.028
Erythroid predominance	0.536	0.109	0.250	1.150
Normal karyotype	0.753	0.076	0.380	1.350
Complex karyotype	1.870	0.013	1.142	3.060
Unfavorable karyotype	1.586	0.019	1.080	2.327
*NPM1* mutations	1.045	0.932	0.382	2.860
*FLT3* mutations	0.839	0.697	0.346	2.031
*RAS* mutations	0.775	0.192	0.418	1.917

## Discussion

We performed this study in order to better understand the clinical, cytogenetic, and prognostic features of patients with AML associated with erythroid predominance. In particular, we defined erythroid predominance as ≥50% bone marrow nucleated cells, as has been done by others, and we compared patients with AML with erythroid predominance to patients with AML without erythroid predominance. We have shown that the presence of erythroid predominance confers a better overall survival in various AML patient groups, with the exception of the therapy-related setting. We further show that patients with AML associated with erythroid predominance have a cytogenetic and molecular profile that differs from patients with AML without erythroid predominance, and more in keeping with patients with high-grade MDS.

Gene mutations in AML pathogenesis have been divided by other into two types; class I and class II. The former affect proliferation whereas the latter affect differentiation. The results of this study, as well as those of previously published studies, suggest that class I mutations that affect myeloblast proliferation, such as *FLT3*, *RAS* and *KIT*, are infrequent in AML associated with erythroid predominance [5], [7], [10], [17]. *NPM1* mutations have been shown to occur at an early stage in stem cells, and *NPM1* mutation is known to affect myeloblast differentiation and play an important role in leukemogenesis. The frequency of *NPM1* mutation reported in AML is highly variable in the literature, ranging from 4% to 100%, presumably attributable, at least in part, to case selection [5], [15], [16], [18], [19], [20]. In this study, we show that the frequency of *NPM1* mutation is extremely low in all types of AML with erythroid predominance, including t-AML, AML-MRC, or AML with a normal karyotype. *JAK2* gene mutations are known to be very common in polycythemia vera, a myeloproliferative neoplasm characterized by expanded erythropoiesis. Although not specifically assessed in this study, others have shown that *JAK2* mutation is rare in AEL [21], [22]. The rarity of class I and II mutations in patients with AML associated with erythroid predominance suggests that the pathogenesis of this group of myeloid neoplasms differs from that of patients with AML without erythroid predominance. We also show that patients with AML associated with erythroid predominance have a lower frequency of an abnormal karyotype and an overall low cytogenetic risk distribution, compared with patients with AML without erythroid predominance. The cytogenetic and molecular genetic features of AML with erythroid predominance therefore are more akin to patients with RAEB in this study.

Erythropoiesis in these cases is prominent, often associated with significant morphologic dysplasia and severe macrocytic anemia, indicating ineffective erythropoiesis, similar to that observed in MDS. In MDS, although not entirely clear, the possible mechanisms involved in ineffective erythropoiesis may be related to mutations or epigenetic dysregulation of a number of important genes, such as *GAT1, RSP14, EVI1 and TEL* [23], [24], [25]. These molecular genetic alterations affect mitochondrial ferritin expression and iron distribution, leading to insensitivity to erythropoietin, increased apoptosis and proliferation, and defective erythroid differentiation. In AML with erythroid predominance, unlike the situation in MDS, the presence of erythroid predominance may be attributable to reduced granulopoiesis or less proliferative granulopoiesis. This is in keeping with the rarity of class I mutations found in this group of diseases. It is noteworthy that the 50% erythroid cutoff used to define AEL is arbitrary and based on historical use of this cutoff in the FAB classification [1]. It is uncertain if a lower or higher percentage of erythroid precursors might be a more effective cutoff in defining a biologically distinctive type of AML associated with erythroid predominance.

Historically, patients with AEL have been thought to have a poor prognosis. However, the definition and classification of AEL have been changed over the years, and particularly so in the most recent WHO classification scheme. Many cases of AML/MDS with erythroid predominance are now classified under other well-defined categories, such as t-AML/MDS, AML with recurrent cytogenetic abnormalities or AML-MRC. In this study, based on the findings in this patient cohort, we question the utility of some aspects of the 2008 WHO classification for the sub-categorization of myeloid neoplasms associated with ≥50% erythroid precursors. The data in this study show that the outcome of patients with therapy-related AML/MDS is generally poor, regardless of the presence or absence of erythroid predominance. Furthermore, the WHO diagnostic categories of RAEB, AEL and AML-MRC do not appear to have prognostic significance in this study, as has been shown by others [7], [9]. The data we present also show that patients with AML with erythroid predominance, including AEL and AML-MRC, had better overall survival than patients with AML without erythroid predominance. In contrast, the prognosis of patients with RAEB appeared to not be affected by the presence or absence of ≥50% erythroid precursors. It is noteworthy that MDS cases with ≥10% myeloblasts would be classified as AEL rather than RAEB-2 in the presence of ≥50% erythroid precursors.

One plausible explanation for the survival advantage of patients with AML associated with erythroid predominance is the overall lower absolute blast count in this group of diseases. Based on the definition, in the presence of ≥50% erythroid precursors, the myeloblast percentage is calculated from the non-erythroid cells, which results in a significantly lower absolute number of myeloblasts in this group of AML cases. However, this explanation is probably too simplistic, or is only a part of the explanation, because the myeloblast count, either enumerated as a percentage of total cells or non-erythroid cells, does not have an impact on clinical outcomes within this group of diseases [5], [7], [9], [26]. Instead, cytogenetic characteristics can stratify these cases into distinctive risk groups: cases with complex cytogenetic abnormalities, especially of chromosomes 5 and/or 7, share biologic and clinical features with t-AML/MDS; whereas, cases with simple cytogenetic abnormalities or diploid karyotypes have a more favorable prognosis. In addition, class I mutations, which are often associated with a poorer prognosis, were infrequent in patients with AML associated with erythroid predominance. For example, the *FLT3* mutation rate of 5.7% found in AML cases with erythroid predominance is much closer to that seen in patients with RAEB than patients with AML without erythroid predominance [11], [27]. In our opinion, it seems likely that cases of AML associated with erythroid predominance have distinctive, but yet unknown molecular genetic abnormalities. Genomic analysis of these cases by high-throughput sequencing may provide clues to pathogenesis.

In summary, the findings in this study provide further evidence that AML associated with erythroid predominance represents a group of diseases with clinical and molecular genetic features that differ from other types of AML. The data presented here suggest that cases of AML with erythroid predominance are clinically, cytogenetically, and at the molecular level much closer to RAEB than they are to cases of AML without erythroid predominance.
